# Visual Light Zones

**DOI:** 10.1177/2041669518781381

**Published:** 2018-06-27

**Authors:** Tatiana Kartashova, Huib de Ridder, Susan F. te Pas, Sylvia C. Pont

**Affiliations:** Perceptual Intelligence Lab, Delft University of Technology, the Netherlands; Experimental Psychology, Utrecht University, the Netherlands; Perceptual Intelligence Lab, Delft University of Technology, the Netherlands

**Keywords:** light, light field, light zones, visual perception, illumination, light properties

## Abstract

In this article, we studied perception of a particular case of light fields that is characterized by a difference in its consistent structure between parts of a scene. In architectural lighting design, such a consistent structure in a part of a light field is called a light zone. First, we explored whether human observers are sensitive to light zones, that is, zones determined primarily by light flow differences, for a natural-looking scene. We found that observers were able to distinguish the light conditions between the zones. The results suggested an effect of light zones’ orientation. Therefore, in Experiment 2, we systematically examined how the orientation of light zones (left-right or front-back) with respect to a viewer influences light inferences in symmetric scenes. We found that observers are quite sensitive to the difference in the light flow of the light zones. In addition, we found that participants showed idiosyncratic behavior, especially for front-back-oriented light zones. Our findings show that observers are sensitive to differences in light field structure between two parts of a scene, which we call *visual light zones*.

## Introduction

Can one make visual estimations of something that can not be seen? Yes, human observers are sensitive to the light field in empty space (Koenderink, Pont, van Doorn, Kappers, & Todd, 2007; Pont, 2009; Schirillo, 2013; Xia, Pont, & Heynderickx, 2014). They can visually fit the intensity, direction, and diffuseness of light on a matte white sphere to a scene basing on appearance of objects in that scene. This sensitivity was named *the visual light field* (Koenderink et al., 2007). Moreover, observers can robustly estimate these light properties throughout an empty space (Kartashova, Sekulovksi, de Ridder, te Pas, & Pont, 2016), and their inferences agree with homogeneous, converging, or diverging superpatterns (van Doorn, Koenderink, Todd, & Wagemans, 2012). The human ability to infer light in (empty!) space is an interesting scientific topic in itself and also relates to questions about interdependency of light, shape, and material perceptions. In this article, we further explore visual light fields by investigating inferences on spatially varying superpatterns, created by variation in illumination over scenes.

Light fields (Gershun, 1939; Mury, Pont, & Koenderink, 2007) in natural scenes can contain uniform, convergent, divergent, rotational, and deformation patterns (Mury, 2009). A uniform pattern is formed by perfectly collimated light (having parallel light rays), for example, direct sunlight. Convergent and divergent patterns are formed by light that focuses in a point or spreads out from a point, respectively. A rotational pattern is formed by light that cycles around a point. Finally, a deformation pattern has a complex flow structure, for example, a saddle. Van Doorn et al. (2012) showed that observers group local shading patterns into global superpatterns that appear to be illuminated in some unitary fashion. They found that observers can perceive uniform, convergent, and divergent patterns but that they are blind to rotational and deformation patterns. The question we address here is whether observers are able to perceive such global superpatterns if a single scene contains two of such patterns.

In the architecture field, such patterns or consistent structures within complex light fields were named *light zones* by Madsen (2007). She introduced the concept of light zones, provided several practical examples and defined them as “(spatial) groupings of the lighting variables (intensity, direction, distribution and colour), which are significant to the space and form-giving characteristics of light.” We note that light zones or the zone system were also introduced in photography by Adams (1948); yet, those zones concern luminance ranges of photographed scenes used for determining optimal film exposure. This is of course closely related to lightness perception and spatial grouping on the basis of intensity, which was already intensely studied (see later). We here study light zones in Madsen’s (2007) sense of spatially segmented parts of a scene that can be grouped on the basis of the structure of the light flow, or, in van Doorn et al.’s words, “global super-patterns that appear to be illuminated in some unitary fashion.” As van Doorn et al., we consider primarily the directional properties of the light field structure, the light flow, for this segmentation. Moreover, we restrict our light zones to flow structures that are uniform/divergent/convergent. Please note that the superpatterns that van Doorn et al. studied were defined in a plane but that they can be easily extrapolated to three-dimensional spaces especially for these simple structures.

Although under different names, the concept of light zones can be found in a number of perceptual studies. The first prominent example is Gilchrist’s (1977) experiment on the perceived lightness of a patch, which depended on its perceived position. Stimuli consisted of two empty spaces, a dark one in the front and a bright one in the back, which were connected with a door opening. When a patch was thought to be in the brightly lit back space (one light zone), its matched lightness was much lower than when it appeared to be in the dim front space (another light zone). Snyder, Doerschner, and Maloney (2005) changed the apparent depth of a probe sphere by manipulating retinal disparity. The sphere could appear to be placed either in a far bright room or in a close dark room, along the observer’s line of sight. Hence, retinal disparity moved the probe between different light zones. This manipulation affected the perceived lightness of the probe. Schirillo (2013) discussed this and other examples in which the lightness and chromaticity of patches were judged in depth planes with different illuminations. He concluded that human observers are able to infer the light in empty space (between illuminated objects). This was tested and confirmed in Koenderink et al.’s (2007) visual light field study. Toscani, Gegenfurtner, and Doerschner (2017) studied the difference in perception of illumination in #thedress scene between observers who see the dress white and gold and those who see it blue and black. The light was probed in front of the dress and in the background. It was found that in the background, there were no differences in chromaticity settings between different perceivers. However, in the foreground, the white-perceivers made bluer settings than blue-perceivers, making the chromatic difference between the front and back of the scene more pronounced. The foreground and background in this case represent two zones with different illuminations. The question remains whether observers are able to spatially segment parts of a scene on the basis of the structure of the light flow, that is, the directional properties of the light.

The study presented in this article was inspired by an experiment using 17th century Dutch paintings (Kartashova, de Ridder, te Pas, Schoemaker, & Pont, 2015), results of which showed a relation between settings consistency and the complexity of light fields in painted scenes. In that experiment, we tested the perception of light on objects and in empty space. Observers were asked to infer light either on volumetric objects cutouts from images of six painted scenes, or in empty space in positions of those cutout objects in the scenes. The consistency of observers’ settings varied greatly between paintings. Four paintings contained seemingly consistently structured light fields (uniform or diverging), or a single light zone. For those paintings, the observers’ settings were rather consistent between conditions (comparing settings for the cutout object with those for the probe in the painting) and within a scene. However, for the other two paintings, the observers’ inferences varied greatly. One of those paintings showed an interior and exterior through a window opening, and the other an interior and back room through a door, thus, both paintings seemed to present two spaces with different light qualities—including its directional properties. The most likely explanation of the inconsistencies in the observers’ settings was the presence of two light zones in both paintings. The observers seemed to interpret the border between the zones idiosyncratically; some inferred two different illuminations in the two parts of the scene, while others made settings as if there was only one light zone. It was an interesting finding; yet, we could not compare the settings of observers to the veridical values in those scenes because they were painted hundreds of years ago.

Our goal in the current study is to investigate the perception of light properties in scenes with two light (direction) zones. We performed two experiments. The first experiment (see Experiment 1 section) had an explorative nature and was done to test whether we could repeat the finding of our former study and to analyze how it is related to the physical light field. We built a model of a natural scene and illuminated it with two configurations of light sources, both creating two light zones. The two configurations were designed to have different orientations with respect to the viewing direction. The visual light field was measured over a grid of points, analyzed, visualized, and compared with the physical light field. In the second experiment (see Experiment 2 section), we focused on the specific question whether the orientation of the light zones (in the picture plane vs. in depth) influences the light inferences. In Experiment 2, we used less probes per scene to allow probing more illumination configurations, and we used a controlled environment. Finally, we discuss the obtained results and propose further directions for research on light zones perception (see Discussion and Conclusion section).

## Experiment 1

The aim of the first experiment was to investigate whether human observers are able to distinguish differences in light properties between physical light zones that were designed to have prominent differences in light directions. We created a scene with two illumination conditions. One condition produced different light zones in the left and in the right part of the scene; another condition created different light zones in the front and in the back parts of the scene. We obtained visual light fields for both illumination conditions by sampling the observers’ light inferences over a grid of positions (for method, see Kartashova et al., 2016).

### Methods

#### Stimuli

**Figure 1.**
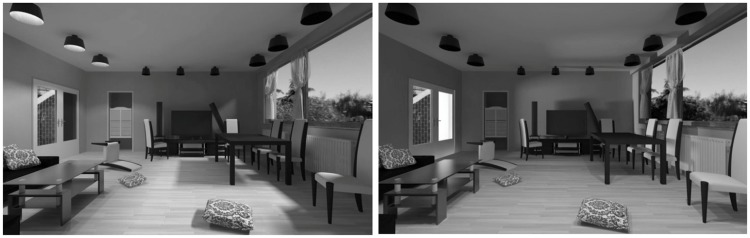
Scenes of Experiment 1. Left is the left-right (LR) condition, and right is the front-back (FB) condition.

We created a model of a scene resembling a living room and illuminated it using two sets of light sources (see Figure 1). In both illumination conditions, the light sources were placed such that approximately half of the scene was in one light zone and approximately half in another. The main difference between the light zones was the light direction. In the first condition, the illumination direction differed between the left and right sides of the scene, creating a left-right (LR) light zones condition. The left side of the scene was illuminated from top-left, with the lamps on the ceiling of the room, and the right part from top-right with the sun shining through the window. The second, front-back (FB) condition had the front part illuminated from the top-front, via lamps above the viewer (not visible in the image), and the back part was illuminated from the left with the light coming through the door in the back of the room. The light of the lamps was simulated by small spherical light sources producing diverging light. The sun- and skylight was simulated by small luminous planes. We rendered images of 1,200 × 900 pixels size with the following settings: linear gamma, mental ray renderer (built-in software for rendering of images and light measurements), minimum 1 sample per pixel, maximum 128 samples per pixel.

#### Setup

To measure the visual light field in the scenes, we used a light probing approach (Kartashova et al., 2016; Koenderink et al., 2007). During the actual measurements, a white matte sphere on a black monopod was superimposed on an image of the scene. Observers could control the direction of the light on the sphere via mouse movements and the intensities of the directed and ambient lights using keyboard buttons. From the observers’ settings, we also extracted the diffuseness of the light, parameterized as 1 minus the ratio between the directed and ambient intensities (Xia, Pont, & Heynderickx, 2016b). The diffuseness can range from fully collimated light (e.g., sunlight) to fully diffuse or Ganzfeld illumination (e.g., light in the mist on a snowy field).

**Figure 2.**
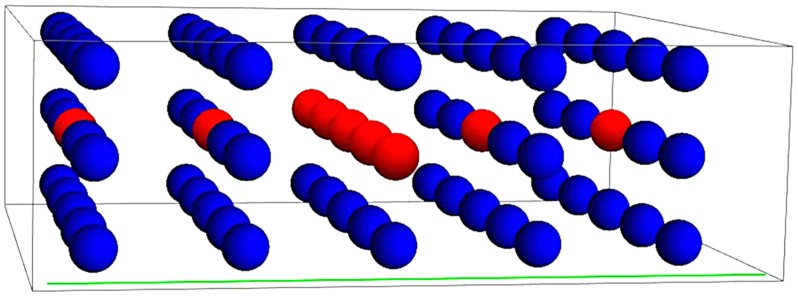
Schematic representation of the measurements positions. The positions of repeated measurements are marked with red. The viewing plane orientation is denoted as a green line.

To define the positions and sizes of the sphere and pole, we created a grid of spheres standing on poles in the model, five spheres in width, five in depth, and three in height. The grid was positioned in the scene such that for both illumination conditions, the vertical middle planes of the grid were on the borders between the light zones (see Figures 2 and 4). The grid was also adjusted such that the poles were always standing on nonoccluded objects to clearly define the spheres positions. For Figure 4, the probes were rendered in the scenes.

For the experiment, we used a high-resolution 15-inch computer screen (2,880 × 1,800 pixels, Retina Display, luminance range from 0.4 cd/m^2^ to 330.8 cd/m^2^). The experiment sequence and controls were developed using the Psychtoolbox library (Brainard, 1997; Pelli, 1997). The light in the room was switched off to avoid illumination interference on the screen. The viewing distance of the observers was fixed at 27 cm from the screen with a chin rest to keep the viewing angle the same as that of the virtual camera used for rendering. The images and probe were presented in grayscale because we wanted primarily to test the influence of the distribution of light. The images, probe, and screen were calibrated linearly.

#### Procedure

At each trial of the experiment, observers were asked to set the illumination on the probe to make it appear as if it belongs to the scene. The experiment consisted of two blocks for the two conditions: LR and FB. Half of the observers started with the LR scene and half with the FB scene to balance the order. In each condition, observers made one setting for each grid position and two additional settings for probes corresponding to the red spheres in Figure 2. Having three repetitions in these points allowed us to compare the spread of the settings within observers across light zones and illumination conditions.

Before the experiment, we explained the procedure, task, and probe controls to the observers. We did not explain the concept of light zones to the observers. To explain the task, we showed a scene resembling a different room that was not used in the experiment, with spheres rendered in it. Then, we performed three trials for training the use of the controls, after which the two parts of the experiment were conducted.

#### Participants

Ten observers participated in this experiment. The participants were naive with respect to the setup and purpose of this experiment. All participants had normal or corrected-to-normal vision. They all gave written, informed consent. All experiments were done in agreement with the Declaration of Helsinki, Dutch Law, local ethical guidelines, and approved by the TUDelft Human Research Ethics Committee.

#### In-scene light measurements

In addition to the psychophysical measurements, we also performed measurements of the light in the modeled scene. For this purpose, we used the Lighting Analysis Assistant tool of the Autodesk 3ds Max system, which allows virtual illuminance measuring. We created a grid of virtual cubic illuminance meters (six illuminance meters on a cube, which allows to measure a first-order approximation to the light field, see Xia, Pont, & Heynderickx, 2016a; Xia et al., 2016b) using a script. We set the measurement cubes to be in the same positions as the probes. The script placed all six meters in the same position, with the sensors facing the positive and negative directions of each axis (see details in Kartashova, de Ridder, te Pas, & Pont, 2018). Then, for each cube, we extracted from the resulting six luminance measurements the intensity, diffuseness, and direction of the light using Cuttle’s (2003, 2013) formulas and interpolated them to obtain a representation of the mathematical first-order structure of the “physical” light field in the scene (Kartashova et al., 2016). Alternative could be estimating the properties from for instance local spherical panoramic images. They are easy to use in rendered scenes, yet would require a spherical harmonics decomposition to compute the properties. Xia, Pont, and Heynderickx (2016a, 2016b) showed that the cubic approach forms a computationally easier approach. Moreover, it can easily be used in real scenes too.

**Figure 3.**
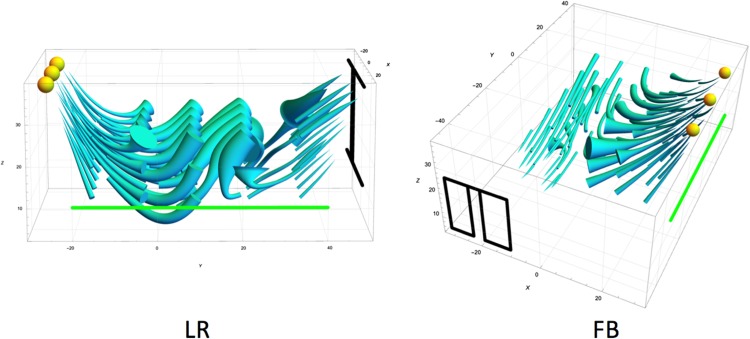
The mathematical first-order structure of the physical light fields for the stimuli used in Experiment 1. Left is the LR (left-right) orientation, and right is the FB (front-back) orientation.

We visualized the light fields as light tubes (see Figure 3). A tube is aligned to the light vectors along its length. A tube’s path is calculated via interpolation methods (see Kartashova et al., 2016; Mury, Pont, & Koenderink, 2009 for the details). The thickness of a tube is inversely proportional to the light intensity. The physical light field in the LR condition contains curved tubes. This happens because in the middle of the measured volume, there is a space that is occluded from all direct light sources, where light arrives mostly through scattering from the floor. Therefore, it is dim (the tubes are rather thick), and the light vector is directed downward (to the floor, a secondary light source). It is clear that the light direction in the left side of the measured volume is very different from the middle and the right side of the volume. There is less curvature in the light field topology for the FB condition because there is almost no space occluded from the direct light sources. As a result, there is a clear division between the light zones, one being in the volume of the light from the door, and the other in the volume of the light from the lamps.

**Figure 4.**
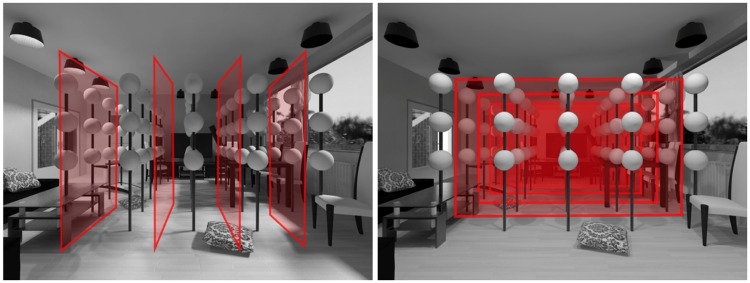
Slicing of the resulting settings. Left is the LR (left-right) condition sliced parallel to the viewing direction, and right is the FB (front-back) condition sliced across the viewing direction.

### Results

From the observers’ settings on the spheres, we obtained psychophysical measurements of the direction, intensities (directed and ambient), and diffuseness of light in the two illumination conditions. The diffuseness *D* was calculated in accordance to the following formulas (Cuttle, 2003, 2013; Kartashova et al., 2016; Xia et al., 2016a, 2016b):

(1) $$E_{(x)}=E_{x+}-E_{x-}$$

(2) $$E_{\text{vector}}=\surd (E_{\left(x\right)}^{2}+E_{\left(y\right)}^{2}+E_{\left(z\right)}^{2})$$

(3) $$∼E_{x}=\frac{E_{x+}+E_{x-}-E_{x\text{vector}}}{2}$$

(4) $$E_{\text{symmetric}}=\frac{∼E_{x}+∼E_{y}+∼E_{z}}{3}$$

(5) $$E_{\text{scalar}}=E_{\text{vector}}/4+E_{\text{symmetric}}$$

(6) $$D=1-(E_{\text{vector}}/E_{\text{scalar}})/4$$

Here, *E*_+_ and *E*_−_ are the illuminance measurements on opposite sides of the cube; *E*_(_*_x_*_)_, *E*_(_*_y_*_)_, and *E*_(_*_z_*_)_ are the light vector components on each axis; *E*_vector_ is the light vector magnitude; *E*_symmetric_ is the symmetric illuminance; and *E*_scalar_ is the mean illuminance in a point. For the observers’ settings, directed and ambient light were taken as the magnitudes of *E*_vector_ and *E*_symmetric_, respectively, after correction for the clipping of intensities on the probe. The diffuseness *D* ranges from 0 (*fully collimated light*) to 1 (*fully diffuse light*).

To analyze the results quantitatively, we grouped the settings according to the positions of the probes. The grid was “sliced” into planes parallel to the border between the light zones (see Figure 4). In the LR condition, the probes were grouped parallel to the viewing direction and in the FB condition parallel to the picture plane. Thus, each of the five groups of data points contained the directional settings of all observers (including the repetitions) on all probes of a slice.

**Figure 5.**
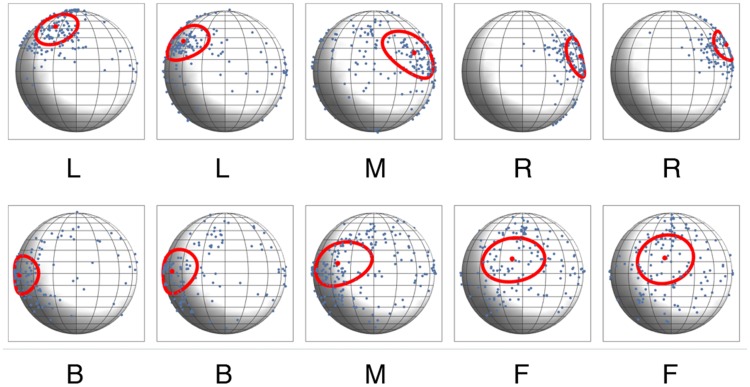
Distribution of settings per slice. The first row shows the results for the LR (left-right) condition, the second row for the FB (front-back) condition. Each sphere represents all the directional settings of all observers on all the spheres of a corresponding slice. The red dots represent the mean directions; the red ellipses represent one standard deviation.

Figure 5 shows the distribution of the directional settings on spheres, with the mean direction represented by a red dot and one standard deviation by red ellipses, for each plane and both conditions. The ellipses’ short and long axes were determined by projecting the data on a plane and calculating standard deviations of the resulting bivariate distributions. It is clear that the settings on the two left spheres in Figure 5 (left light zone for LR, and back light zone for FB) are dramatically different from the settings on the two right spheres (right light zone for LR, and front light zone for FB). The angular differences between the means of the different light zones are large: from 82° to 111° for LR and from 49° to 61° for FB. In contrast, the differences for planes in the same light zones are small: 24° and 12° for LR, and 4° and 8° for FB. This suggests that observers were on average able to distinguish the light zones. However, the data show quite some variation, and therefore we now analyze individual results in detail.

**Figure 6.**
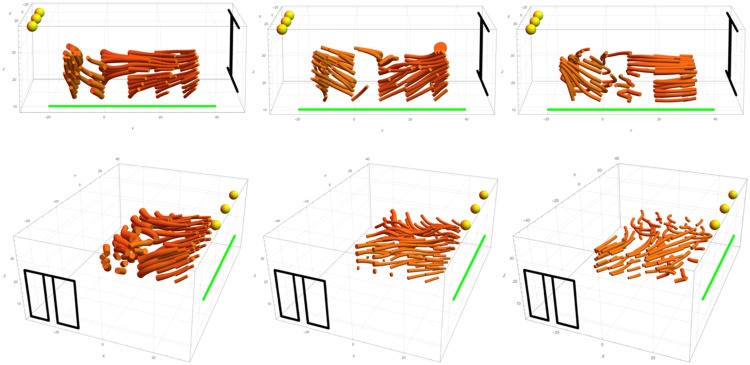
Pairs of visual light fields of three representative observers. The first row represents results for the LR (left-right) condition, the second row for the FB (front-back) condition. The green line denotes the picture plane. Yellow spheres represent the lamps. Black lines show the positions of the window and the door. The visualizations of the LR conditions have a rather clear border between the light zones, whereas for the FB conditions, the visual light fields vary idiosyncratically.

Having the observers’ settings for a grid of points allowed us to reconstruct the visual light fields via interpolation (Kartashova et al., 2016; Mury et al., 2009); see Figure 6 for three representative cases per condition. Generally, in the LR condition, there is an apparent distinction between the light zones with the border slightly varying from one observer to another, but roughly in the middle of the measured volume (see Figure 6, top row). For none of the observers, we found curved light tubes in the center of the scene, as in the physical light field (see Figure 3). This confirms our previous finding that human observers ignore subtle variations in physical light fields (Kartashova et al., 2016). The visual light fields in the FB condition differed from one observer to another (see Figure 6, bottom row). In the left visualization, the tubes in the back of the room seem to flow to the door, whereas the tubes in the rest of the room point to the lamps in front; in the second figure, all the tubes point to the lamps; and in the third, most of the tubes point to the door.

**Figure 7.**
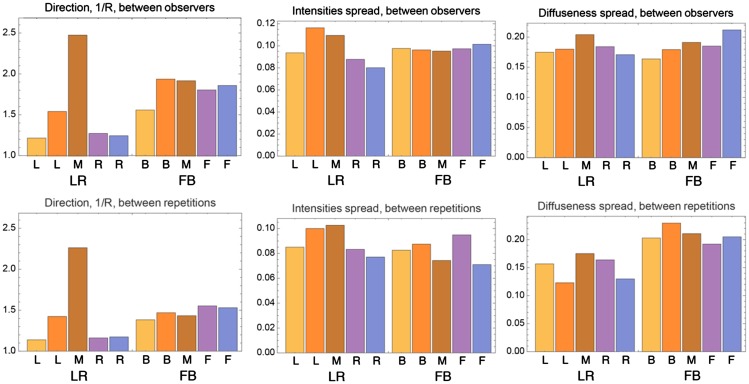
Spreads of settings between observers in the first row and between repetitions in the second row. The first column concerns the spread in the directional settings, the second column in the intensity settings, and the third column in the diffuseness settings. For the bar charts between observers, each bar represents the spread between all observers’ settings in one slice of the grid. For the bar charts between repetitions, each bar represents an average of the spreads between observers’ repetitions for each probe. LR = left-right; FB = front-back.

We analyzed the spreads of the data between and within observers (see Figure 7). As a measure of the spread of the directional settings, we took the dispersion 1/*R*, where *R* was the length of the vector summation of the settings represented as unit vectors divided by the number of measurements (Leong & Carlile, 1998). *R* has the highest value (namely 1) when all the vectors point in the same direction, it decreases with increasing spread, and it is smallest (namely 0) for data that are uniformly spread over all directions. So, 1/*R* ranges from 1 to infinity. The motivation of using such method is that the data have a spherical nature, and therefore, such a spherical analysis is better suited than splitting the data into two angles. For the interobserver spread of the directional settings, represented in the first column and row of Figure 7, we calculated the dispersion 1/*R* for all settings of all observers on all spheres of a slice, including the repetitions (thus, the same data as we grouped in Figure 5 for calculating means and ellipses). For the LR light condition (left half of the graph), the dispersion peaks on the middle plane and otherwise is relatively low. The dispersions in the FB condition (right half of the graph) seem more uniform across the slices than in the LR condition and on average higher. We did not calculate the significance of the differences between the data for the slices because we did not have a tool at hand for the analysis of such spherical data. The dispersion between repetitions (within observers) was calculated using the repeated settings data for five probes. The five probes were selected from the nine repetition probes such that two of the probes lay in one light zone, two in the other light zone, and one in the center of the grid. The dispersions between repetitions showed a similar pattern as those between observers. Moreover, the values of 1/*R* between repetitions or within observers seemed, overall, somewhat smaller than between observers.

To compare the intensity settings, we calculated the scalar illuminance (see Equation 5). For diffuseness, we used the normalized diffuseness formula of Xia (Cuttle, 2003; Xia et al., 2016a; see Equation 6). We took the standard deviation as a measure of the spread of the intensity and diffuseness (see Figure 7). We found that the spreads for the intensities (ranging from 0 to 1) are always between 0.07 and 0.12 and for the diffuseness (ranging from 0 to 1) between 0.10 and 0.25, and we did not find significant differences for these data. Moreover, again the values between repetitions or within observers seemed overall somewhat smaller than between observers.

Summarizing the results, the observers were able to distinguish the illumination differences between the light zones. In addition, we found trends in the data, indicating that there might be idiosyncratic differences as well as differences between the light conditions: In the LR condition, the results seemed to be consistent, except the plane between the light zones, whereas in the FB condition, the spread values seemed on average higher than in the LR condition. The question remains whether these differences between the conditions were genuine or that they were evoked by other stimulus properties. The positions of light sources were different between the conditions, with respect to the scene geometry. Specifically, in the LR condition, the light sources were on two opposite sides of the scene, and in the FB condition on two perpendicular sides. In addition, in the LR condition, the observers could see the lamps and not in the FB condition. Finally, the scene was not symmetric.

We performed a second experiment to study if the trends in the findings were indeed caused by the light zones’ orientations. In Experiment 2, we eliminated all the listed interfering differences between conditions.

## Experiment 2

The goal of this experiment was to systematically investigate light perception for LR and FB orientations of light zones. A rotationally symmetric scene was illuminated with three configurations of light sources, creating physical light zones. Viewing each illumination condition from two perpendicular directions allowed us to test LR and FB orientations of the light zones while keeping the actual light and geometry in the scene constant.

### Methods

#### Stimuli

**Figure 8.**
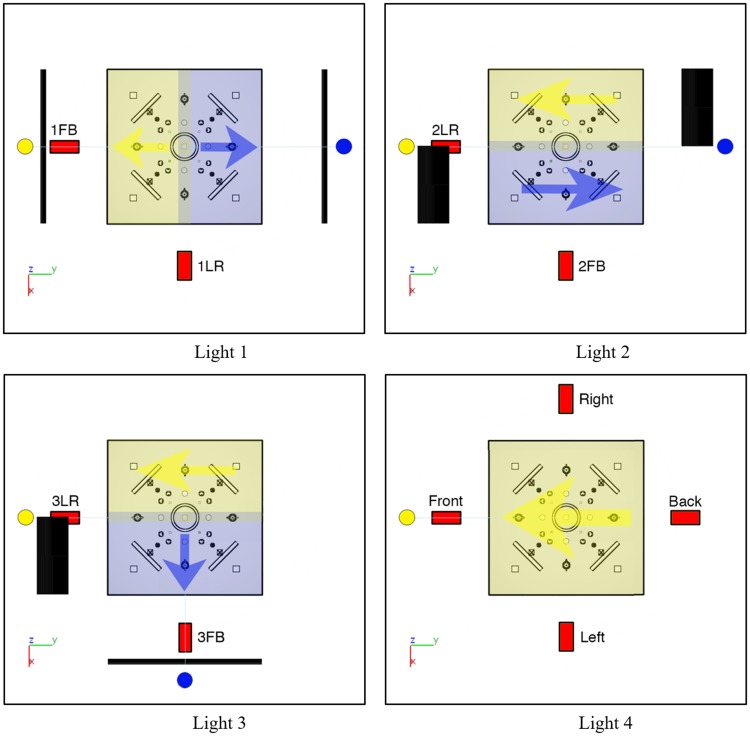
Schematic representation of the tested conditions and views. Each image represents the top view of the scene and light sources (the distances to light sources and shades are not proportional). Red rectangles represent the cameras, labeled according to the resulting scene image. Yellow and blue circles represent the light sources. Black bars show the shades, which partially occluded the light, so that the light source illuminated only the closest half of the scene. Black rectangles show the shades that completely occluded the light on a half of the scene. Yellow and blue arrows show the approximate light orientation (as a vector, pointing toward the source) in the zones of corresponding colors.

**Figure 9.**
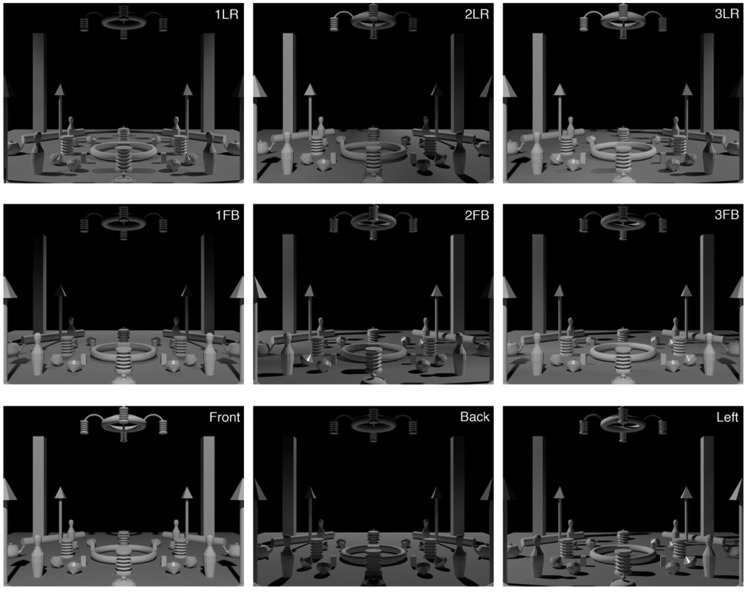
Test images. The first two rows contain the conditions with two light zones. The first row shows the LR orientations, the second row the FB orientations. The third row shows the single light zone condition from the left, front, and back (the right is not shown here because that is exactly mirrored to the left case).

The constructed scene contained a set of simple shapes that were placed and adjusted such that the scene was geometrically symmetric with respect to 90° rotations (see Figures 8 and 9). The objects of the scene were chosen to induce a variety of light cues: shading, shadows, highlights, and interreflections. All objects were white, and most of the objects, except four spinners, were matte. The spinners were glossy. The ground plane was midgray, and the background was black. The scene was created and rendered in the same software as in Experiment 1.

For each illumination condition, the light sources were positioned at a 45° elevation with respect to the center of the scene and the ground plane. For the first three conditions, we illuminated the scene with two identical small spherical light sources (see Figures 8 and 9). The conditions were viewed from two directions, such that from one viewpoint the light zones were on the left and on the right side of the scene (1LR, 2LR, and 3LR), and from the other viewpoint in the front and in the back of the scene (1FB, 2FB, and 3FB). We modulated the positions of the light sources and shades to create light zones differing in average light direction. Conditions 1 and 2 both have identical positions of the light sources at opposite sides of the scene (see Figure 8, first row). In Condition 1, the shades partially occluded the light so that the light source illuminated only the closer half of the scene. In Condition 2, the shades completely occluded the light on half of the scene. The placing and orientation of shades in these two conditions create light zones with complementary directions. As a result, in 1LR, the scene is illuminated from the left and the right (similarly to LR condition of Experiment 1), whereas in 2LR from the front and back. Condition 3 was selected to test a condition that is similar to the FB condition of Experiment 1. In this condition, we used the combination of the shades used in Conditions 1 and 2. The fourth condition contained a single light source, identical to the sources of previous conditions, creating a quite homogeneous single light zone. This single light zone condition was created to have baseline settings for each light direction. It was viewed from each side of the scene (front, right, back, and left). Thus, altogether, we created 10 test images, 6 with two light zones and 4 with a single light zone (see Figure 9).

#### Setup and procedure

**Figure 10.**
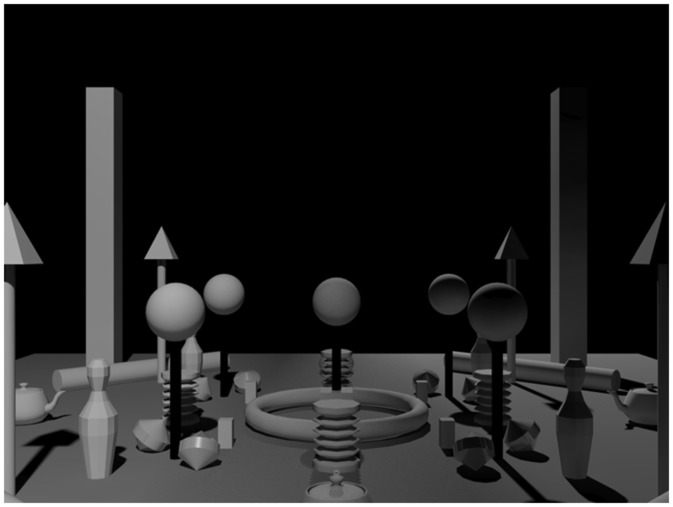
White spheres illustrating the positions of probes (for 2LR (left-right), with veridical probe illuminations). Each scene contained five probes, four in each quadrant of the scene and one in the center of the scene. Only one probe was shown at a time. A probe did not produce a shadow in the trials.

The setup in Experiment 2 was the same as in Experiment 1. Each test image contained five probes, one in each quadrant of the scene and one in the center (see Figure 10)—but during the experiment, only one probe was shown per trial. Five probes do not suffice to model the global light field but allowed us to include more scenes in the experiment and systematically test the effects of light directions *and* viewing directions. The adjustments were repeated three times for each probe and each test image. Thus, 10 test images × 5 probes × 3 repetitions constituted 150 trials, of which we randomized the order of presentation.

Before the experiment, we explained the procedure, task, and probe controls to the observers. We did not explain the concept light zones to the observers. To explain the task, we showed two illuminations of the scene, which were not used in the experiment, with spheres rendered in it. Then, we performed three trials for training the use of the controls and the experiment trials.

As in Experiment 1, we also conducted “physical” light measurements in the scene at the positions of the probes for each illumination condition.

#### Participants

Ten observers (different observers than who participated in Experiment 1) participated in this experiment. The participants were naive with respect to the setup and purpose of this experiment. All participants had normal or corrected-to-normal vision. They all gave written, informed consent. All experiments were done in agreement with the Declaration of Helsinki, Dutch Law, local ethical guidelines, and approved by the TUDelft Human Research Ethics Committee.

### Results

The results of Experiment 2 consisted of observers’ repeated settings on the five probes for the 10 test images, 6 of which were pairs of light zones conditions, and the remaining 4 considered the single light source condition. We made several comparisons of the results for the direction settings. We arranged the results for each test image in three groups, with the first group representing the settings on the two probes of the first light zone (L for LR and F for FB), the second group of the single middle probe, and the third group of the two probes of the second light zone (*R* for LR and B for FB). For the single light source condition, the data were grouped into left, middle probe, and right parts of the scene with regard to the viewing direction.

**Figure 11.**
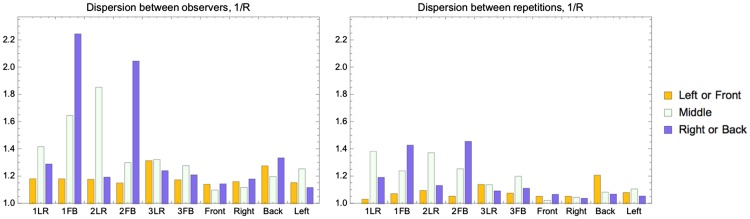
Inter- and intraobservers’ variability of the directions of the settings (parameterized via the dispersion).

We parameterized the directional variability via the dispersion 1/*R* as described in Experiment 1. Figure 11 shows that the dispersions are overall quite low and that they are significantly lower between repetitions than between observers (paired *t* test: *t* = 3.04, *p* = .002 < .05). There are clear peaks for the B zones of 1FB and 2FB, both between observers and between repetitions. The peaks are even higher than on the middle sphere, where high dispersion could be expected because this probe is on the border between the light zones.

**Figure 12.**
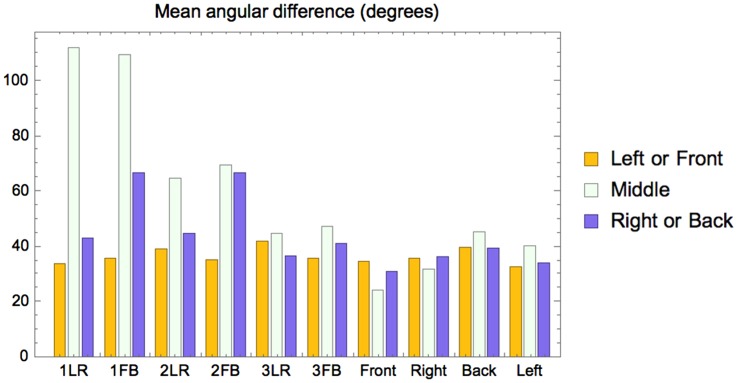
Deviations with respect to the veridical average light direction (light vector) as a function of condition.

For comparison with the physical light directions for each probe, we calculated the mean angular differences between the veridical and the observers’ inferred light directions. These deviations from veridical again showed peaks for the B zones of 1FB and 2FB. We tested if deviations from the veridical values for back light zones are significantly different from deviations from the veridical values for front light zones. We made paired *t* tests of these groups for 1FB and 2FB. The differences are significant (1FB: *t* = − 4.91, *p* = 2.4 × 10^−6^; 2FB: *t* = 4.46, *p* = .000013). The large deviation from veridical that we find for the middle sphere of Condition 1 (middle bar of 1LR and 1FB in the Figure 12) is due to the physical light direction being from below (light vector pointing down), while the observers inferred it to be from above.

**Figure 13.**
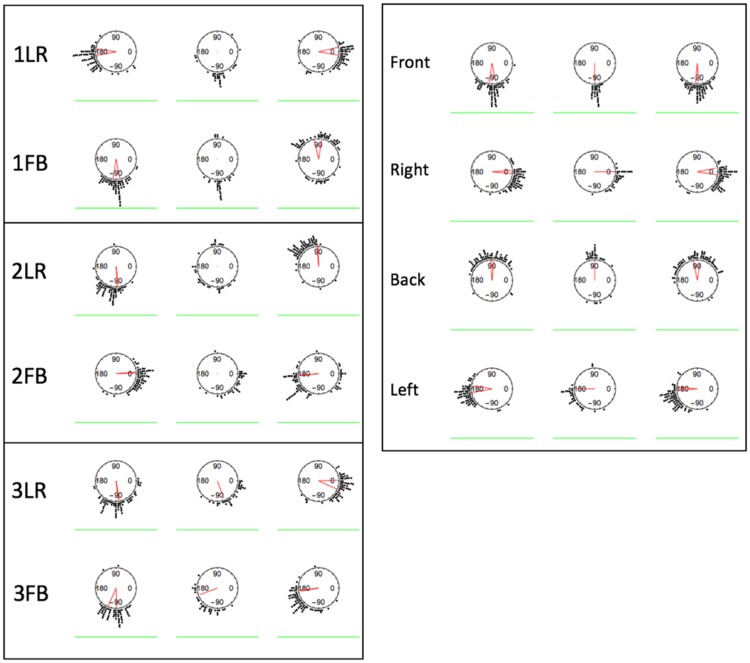
Top view circular histograms of direction settings. Settings are grouped such that the left histogram of a test image shows the settings made on the two probes in the left or front light zone (for LR or FB, respectively), the middle histogram shows the settings on the middle sphere, and the right histogram shows the settings made on the two probes in the right or back light zone (for LR or FB, respectively). The red lines in the centers of the circles show the veridical light directions (red dots instead of lines mean that veridical light vector points straight up or straight down). The green line represents the picture plane.

We also analyzed the distributions of the directional settings. Because all the light sources were positioned on the same height (specifically, making a 45° angle between light source, middle of the scene and the scene floor plane), the differences in their positions could be specified in the XY-plane/a top view. Figure 13 shows the circular histograms of the settings for the data of all test images, with the data grouped in three clusters as above. The red lines in the centers of the circles show the veridical light directions (red dots instead of lines mean that the veridical light vector points straight up or straight down). First, it is evident that the majority of the settings are close to the veridical directions. Thus, observers were well able to distinguish the light zones. If we take a closer look, we can see that for the back light zone of 1FB and 2FB, there is a number of settings in the direction opposite to the veridical, whereas this is not the case for the LR conditions. It appears from the plots that observers often made settings on the probe in the back light zone opposite to the actual illumination in that light zone, but in accordance to the illumination in the front light zone. In addition, for these images, the middle probe settings appear the same as those of the front probe, which is not the case for the LR conditions.

As we explained in the Methods section, the two test images of the same light condition differed only in the direction of view. Thus, if the settings would depend only on light condition and not on viewing direction, the settings for the LR and FB cases should be the same up to a 90° rotation. For example, the settings in the L light zone of 1LR should be the same as in the F light zone of 1FB, after a 90° correction. We tested the significance of the differences between pairs of directional settings (top view, as presented in Figure 13) after 90° corrections. To this aim, we used Watson’s U^2^ test (code by Mégevand [https://github.com/pierremegevand/watsons_u2] based on equations by Zar, 1999) for circular distributions. First, we compared the settings between the couples of light zones under the same illumination (see Table 1). For example, L of 1LR versus F of 1FB, and L of 2LR versus B of 2FB. We found (see Table 1) that the front light zones of all FB conditions were not significantly different from the corresponding zones of the LR conditions, except for light Condition 2. However, the back light zones of all FB conditions were significantly different from the corresponding zones of the LR conditions.

**Table 1.** Summary and *p* Values of the Light Zones Comparisons.

Comparison of settings between light zones	Front vs. (rotated) corresponding zone	Back vs. (rotated) corresponding zone
Light 1	.067 (nonsignificant difference)	<.001 (significant difference)
Light 2	<.001 (significant difference)	<.001 (significant difference)
Light 3	.433 (nonsignificant difference)	<.001 (significant difference)

Single light source settings: 4R and (mirrored) 4L: *p* = .117 (not significantly different); 4F and (mirrored) 4B: *p* < .001 (significantly different).

For the single light source condition, the settings were compared for the pairs 4L versus 4R and 4F versus 4B. We compared the pairs after flipping. The settings of 4L did not differ significantly from the (mirrored) settings of 4R. There was a significant difference between settings 4F and (mirrored) settings for 4B.

## Discussion and Conclusion

We investigated whether human observers can distinguish light zones that are determined by a difference in overall light direction. In the first experiment, we measured and analyzed visual light fields for two illumination conditions. In the second experiment, we created highly controlled stimuli to systematically investigate the influence of light zones’ orientations. Specifically, we tested illumination perception in LR and FB orientations of light zones.

The distributions, spreads, and visualizations of the first experiment’s settings show that observers were able to distinguish the illumination differences between the light zones. This sensitivity we call *visual light zones.* There seemed to be a trend, suggesting that interobserver spreads were larger than intraobserver spreads, especially for the FB condition. The results of the second experiment confirmed the existence of visual light zones. In Experiment 2, we again found that interobserver spreads were larger than intraobserver spreads, indicating idiosyncratic behavior. In addition, we found a difference between LR and FB orientations of light zones. In particular, the observers often made the settings in the back light zone of the FB conditions in accordance to the illumination in the front light zone of those conditions.

One of the reasons of the differences in the settings between the light zones’ orientations might be the visibility of light cues, for example, shadows, shading, and highlights (Boyaci, Doerschner, & Maloney, 2006; Koenderink, van Doorn, Kappers, te Pas, & Pont, 2003; Lopez-Moreno, Sundstedt, Sangorrin, & Gutierrez, 2010; O’Shea, Agrawala, & Banks, 2010; Pas, Pont, Dalmaijer, & Hooge, 2017; Ripamonti et al., 2004; Xia et al., 2014, 2017). In the LR orientation, each light zone took half of the picture plane, and therefore, it was likely that in both light zones, the cues were approximately equally well visible. In the FB condition, light cues in the back part might be less obvious than those in the front because of occlusions and perspective/scaling. Boyaci et al. (2006) and Xia et al. (2017) demonstrated how in the presence of less (articulated) cues the veridicality of participants’ settings declined.

There are still many unanswered questions about light zones. One of the concerns is how the grouping of such patterns happens. Illuminance flow patterns (Pont, van Doorn, Wijntjes, & Koenderink, 2015) are a probable candidate as a cue for direction-based zones. The exact mechanisms are subject of further studies. In addition, it needs to be studied how intensity, direction, diffuseness, and color interact in the perception of light zones. Furthermore, grouping light zones can be done at different scales of analysis. This scale (the dimensions of the light zones with respect to the scene) determines the relative size and the number of light zones in natural (usually complex) light fields. It defines, for instance, whether relatively small parts of a scene are determined to be a separate light zone or part of a larger one. In Koenderink et al.’s (2007) visual light field study, we find an interesting example. They tested a position in their spotlight condition where the probe was in a relatively small shadow volume that was cast by one of the objects (penguins). Only one of eight observers inferred the probe to be in the shadow, but others adjusted the illumination on the probe as if it was in the spotlight. It might well be the case that visual light zones concern a rather coarse-scale analysis that neglects such fine-scale light variations. Further studies are needed to fully understand these mechanisms.

Our findings might help to understand the structure of the visual light field, which can contribute to several applied areas. In computer graphics, volume zoning was introduced for the case of participating media, for example, dust or fog (Rushmeier & Torrance, 1987). We believe that research on light zones could be useful for automated creation of light probes (Chajdas, Weis, & Westermann, 2011). It would be also interesting to test the veridicality of perception of light on objects moving through light zones, extending the existing studies of lightness estimations of an object moving though differently illuminated areas (Toscani, Zdravković, & Gegenfurtner, 2016; Zdravković, 2008). Would observers notice it if a change of illumination on a moving object does not match the variations of the light in a space through which it is moving? How (in)sensitive are we to such changes? Can we simplify implementations by modeling complex natural light fields in a segmented model containing only a few zones with simple uniform/divergent/convergent light flows? Light zones are regularly used in architecture and light design. Madsen (2007) coined the concept and provided a number of practical architectural examples. There is a continuing interest in the topic from researchers in the field of architecture and lighting (Lindh, 2013; Trisha & Imran, 2014). We sharpened the definition of light zones and provided an approach for measuring and evaluating their perception. Finally, our results confirmed the existence of visual light zones.
